# Physical activity patterns and cognitive function in elderly women: a cross-sectional study from NHANES 2011–2014

**DOI:** 10.3389/fnagi.2024.1407423

**Published:** 2024-06-12

**Authors:** Junyu Wu, Peng Qiu, Meihan Liu, Weiqiang Yu, Min Li, Youqiang Li

**Affiliations:** ^1^School of Physical Education, Shanghai University of Sport, Shanghai, China; ^2^Department of Rehabilitation Medicine, The First Affiliated Hospital of Wenzhou Medical University, Wenzhou, Zhejiang, China; ^3^College of Professional Studies, Northeastern University, Boston, MA, United States; ^4^Sport Science School, Beijing Sport University, Beijing, China

**Keywords:** physical activity, weekend warrior, regular exercise, cognitive function, elderly women, NHANES

## Abstract

**Background:**

Amid the backdrop of global aging, the increasing prevalence of cognitive decline among the elderly, particularly within the female demographic, represents a considerable public health concern. Physical activity (PA) is recognized as an effective non-pharmacological intervention for mitigating cognitive decline in older adults. However, the relationship between different PA patterns and cognitive function (CF) in elderly women remains unclear.

**Methods:**

This study utilized data from National Health and Nutrition Examination Survey (NHANES) 2011–2014 to investigate the relationships between PA, PA patterns [inactive, Weekend Warrior (WW), and Regular Exercise (RE)], and PA intensity with CF in elderly women. Multivariate regression analysis served as the primary analytical method.

**Results:**

There was a significant positive correlation between PA and CF among elderly women (β-PA: 0.003, 95% CI: 0.000–0.006, *P* = 0.03143). Additionally, WW and RE activity patterns were associated with markedly better cognitive performance compared to the inactive group (β-WW: 0.451, 95% CI: 0.216–0.685, *P* = 0.00017; β-RE: 0.153, 95% CI: 0.085–0.221, *P* = 0.00001). Furthermore, our results indicate a progressive increase in CF with increasing PA intensity (β-MPA- dominated: 0.16, 95% CI: 0.02–0.09, *P* = 0.0208; β-VPA-dominated: 0.21, 95% CI: 0.09–0.34, *P* = 0.0011; β-Total VPA: 0.31, 95% CI: −0.01–0.63, *P* = 0.0566).

**Conclusion:**

Our study confirms a positive association between PA and CF in elderly women, with even intermittent but intensive PA models like WW being correlated with improved CF. These findings underscore the significant role that varying intensities and patterns of PA play in promoting cognitive health among older age groups, highlighting the need for adaptable PA strategies in public health initiatives targeting this population.

## 1 Introduction

Cognitive function (CF) refers to the mental processes involved in the acquisition, processing, storage, and retrieval of information, including memory, attention, executive functions, language abilities, and Perception. Cognitive decline profoundly impacts the elderly (age > 60 years), leading to increased dependency, elevated healthcare costs, and reduced quality of life, thus highlighting its significance as a paramount public health challenge (Vos et al., 2020; Lastuka et al., 2024; Monteiro et al., 2024). Alzheimer's disease has been reported as the sixth leading cause of death in the United States (Ross et al., 2021; To, 2023). Currently, there are 6.7 million Americans aged 65 and older living with Alzheimer's, with projections suggesting an increase to 13.8 million by 2060 (Ross et al., 2021). Notably, cognitive decline is more prevalent and severe among elderly females, such as mild cognitive impairment (Liu et al., 2024), Alzheimer's disease (To, 2023). This demographic trend underscores the urgent need for innovative and immediate interventions aimed at the prevention of cognitive decline specifically in elderly women, marking a pivotal focus for healthcare research and innovation.

Physical activity (PA) is defined as any bodily movement produced by skeletal muscles that results in energy expenditure, which is widely recognized as a pivotal factor in promoting cognitive health and mitigating the risks associated with cognitive decline (Flicker et al., 2011; Sofi et al., 2011; Sobol et al., 2016; Sabia et al., 2017; Lamb et al., 2018). Extensive research corroborates the positive correlation between regular physical exercise and improved cognitive function (CF) across various age groups, particularly in the elderly (Nguyen et al., 2023; Zhang et al., 2023). Mechanistically, exercise induces a plethora of physiological benefits, including enhanced blood flow to the brain (Ahlskog et al., 2011), increased growth factor levels that facilitate neurogenesis (Ahlskog, 2011), and improved mood and sleep (Jiang et al., 2024; Soini et al., 2024), all of which are conducive to cognitive wellbeing. However, the relationship between different patterns of PA, such as the “weekend warrior” (WW) model (characterized by concentrated bouts of exercise during limited days) vs. regular exercise (RE), consistent exercise routines, and their respective impacts on the cognitive health of elderly women remains underexplored (Öztürk et al., 2023). This gap in knowledge signifies a critical area of investigation, as understanding the differential effects of these exercise patterns could inform more personalized and effective approaches to preventing cognitive decline in this high-risk population.

Building on the established importance of PA for cognitive health, this study aims to delve into the specific impacts of varying exercise patterns, particularly contrasting the WW with RE. Our analysis utilizes the comprehensive data from the National Health and Nutrition Examination Survey (NHANES) for the years 2011–2014. The rich detail of the NHANES dataset, which includes extensive demographic, lifestyle, and health-related information, provides a robust basis for assessing how distinct patterns of physical activity influence cognitive outcomes. This study aims to identify the most effective PA patterns for preserving cognitive health in the elderly female population, thereby informing the development of targeted, evidence-based public health strategies and personalized preventive measures.

## 2 Methods

### 2.1 NHANES and participants

The NHANES is a program of studies designed to assess the health and nutritional status of adults and children in the United States. This survey combines interviews and physical examinations to collect and analyze health-related data from a nationally representative sample, providing insights into various health parameters, including lifestyle, fitness, diet, and mental health (Marco et al., 2022).

**Figure 1:**
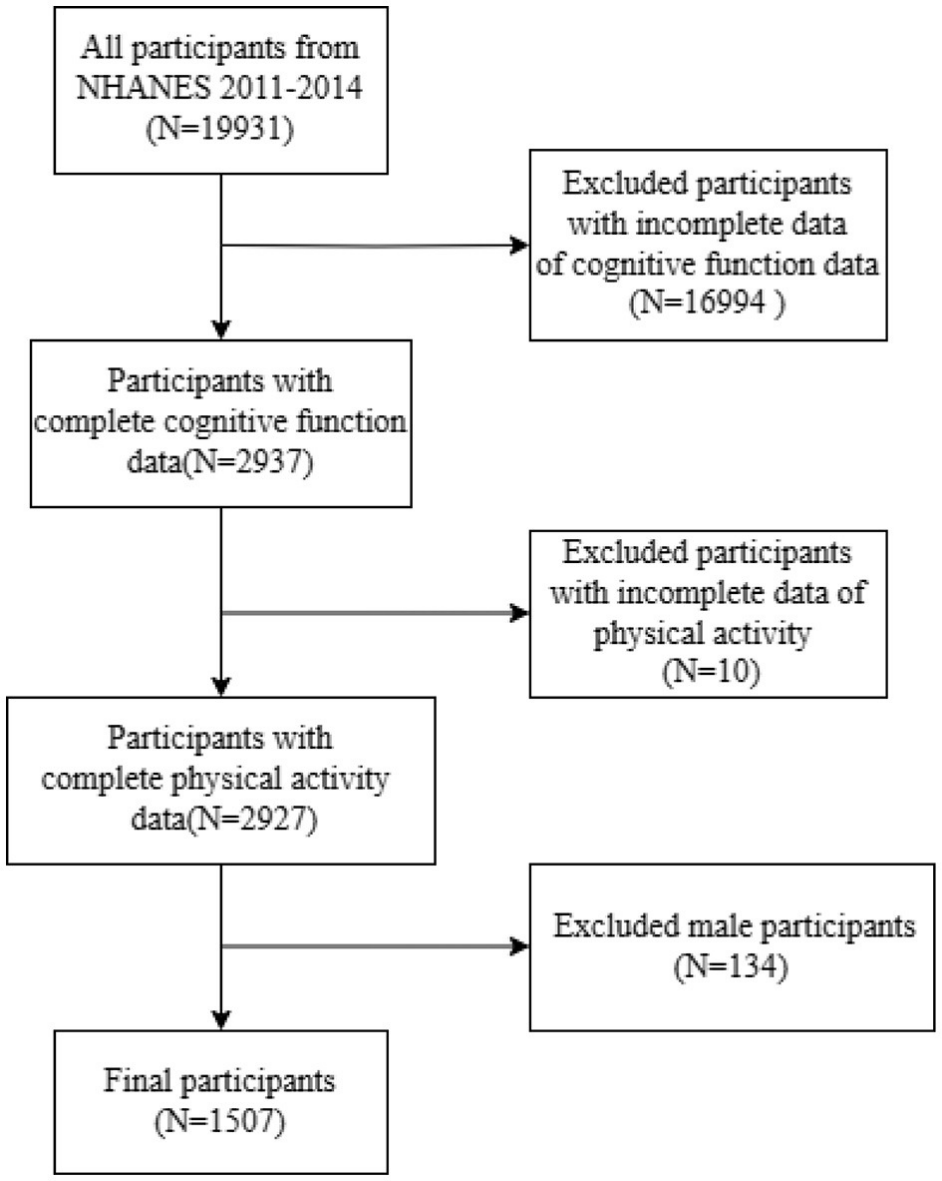
Flowchart of participants inclusion exclusion.

In our study, we focused on data from the NHANES specifically from the years 2011 to 2014, as this was the period during which CF data for elderly participants were collected. The process of participant inclusion and exclusion is illustrated in Figure 1. Initially, the cohort consisted of 19,931 participants. We first excluded individuals with incomplete CF data, leaving 2,937 subjects. Subsequently, we removed participants lacking complete PA data, which further narrowed the field to 2,927 individuals. Finally, we excluded male participants from this group, resulting in a final sample size of 1,507 participants for our study.

### 2.2 Physical activity

In our investigation, participant-reported PA levels were measured using the Global Physical Activity Questionnaire (GPAQ), focusing specifically on the frequency and duration of activities conducted during leisure time (Cleland et al., 2014). This method is adapted to accurately capture instances of moderate (MPA) and vigorous (VPA) physical activities that last for a minimum of 10 continuous minutes. We calculated weekly leisure-time PA by aggregating the total minutes engaged in MPA and VPA, adjusting for intensity differences by equating 1 min of VPA to 2 min of MPA (Piercy et al., 2018; Lei et al., 2024). Furthermore, PA was analyzed both as a continuous variable and categorical variable. We differentiated total PA into three primary patterns: “Inactive” for those accumulating < 150 min of total PA per week (Bull et al., 2020); WW for individuals meeting or exceeding 150 min of total PA weekly within one or two sessions; and RE for those distributing the same or greater amount of PA across more than two sessions weekly (Lei et al., 2024). Additionally, we introduced a refined classification to assess PA intensity more precisely, segmenting it into four categories: “MPA-Total” where all activity is of moderate intensity, “MPA-dominated” for activities predominantly of moderate intensity but including some vigorous activity, “VPA-dominated” where vigorous activity exceeds moderate intensity, and “VPA-Total” for exclusively vigorous activity. This stratification allows for a more detailed exploration of the impact of different PA intensities and frequencies on cognitive health outcomes in the elderly female cohort.

### 2.3 Cognitive function

In our study, CF of participants was assessed using a suite of standardized cognitive tests derived from the NHANES 2011–2014 dataset. This evaluation encompassed the Consortium to Establish a Registry for Alzheimer's Disease Word List (CERAD-WL), which measures immediate recall (IR) across three attempts and delayed recall (DR) after a predetermined period, effectively gauging short-term memory and the ability to retain learned information (Morris et al., 1993). Additionally, cognitive flexibility and semantic memory were evaluated using the Animal Fluency (AF) test, which requires participants to name as many animals as possible within 1 min, testing their rapid semantic retrieval abilities (Canning et al., 2004; Brody et al., 2019). Executive function and processing speed were examined through the Digit Symbol Substitution Test (DSST), involving a task that assesses attention, visual-motor coordination, and cognitive speed by requiring participants to match symbols with numbers (Amaresha et al., 2014; Jaeger, 2018).

For analytical purposes, Z-scores were computed for each cognitive test to standardize the results across different cognitive domains, facilitating comparative analysis (Wilson et al., 2002). A comprehensive CF score was then derived by calculating the mean of these Z-scores, providing a singular, overarching metric of cognitive performance in our study's participants (Wilson et al., 2002; Chu et al., 2022). This composite measure serves to present a holistic view of an individual's cognitive status, integrating various CF into a unified framework.

### 2.4 Covariates

In our study, a meticulously curated set of covariates was employed to mitigate potential confounding influences on the relationship between PA and CF, grounded on insights from prior research literature (Jia et al., 2024; Ren et al., 2024; Zuo et al., 2024). These covariates included critical demographic attributes such as gender, race, age, and educational attainment, alongside socio-economic indicators exemplified by the Poverty Income Ratio (PIR). Additionally, we considered health and lifestyle factors, including body mass index (BMI), frequency of alcohol consumption, waist circumference, and the presence of sleep disorders. Furthermore, prevalent health conditions such as smoking status, diabetes, and depressive symptoms were also integrated. This strategic inclusion of covariates was designed to ensure a comprehensive control of factors acknowledged to affect cognitive health, thereby augmenting the integrity and robustness of our analytical outcomes.

### 2.5 Statistical analysis

In our study, statistical analyses were performed using R software version 4.2.3 and EmpowerStats version 2.0, with a *p* < 0.05 deemed to indicate statistical significance. In terms of methodology, we primarily employed multivariate linear regression analyses to investigate the relationship between PA, PA patterns, and PA intensity with CF among elderly women, treating PA as both a continuous and categorical variable. Additionally, we utilized smooth curve fitting techniques to explore the nonlinear relationships between PA and CF. To ensure the robustness of our results, subgroup analyses were conducted to examine the relationship between these variables further.

## 3 Results

### 3.1 Basic information for participants

As delineated in Table 1, significant disparities were observed among elderly female participants engaging in different PA patterns (Inactive, WW, RE) in terms of sociodemographic characteristics, including age, educational level, and PIR. Furthermore, variations in lifestyle factors such as sedentary time, frequency of alcohol consumption, and sleep duration were also significantly different across the distinct PA patterns. Lastly, health status indicators, including combined handgrip strength, BMI, waist circumference, depressive symptoms, and diabetes prevalence, exhibited significant discrepancies among participants adhering to different PA regimens.

**Table 1:** Basic information for participants.

	**Inactive (*N =* 814)**	**WW (*N =* 30)**	**RE (*N =* 663)**	***P*-value**
**Continuous variables (M** ±**SD)**				
Age (year)	70.36 ± 6.81	72.02 ± 6.38	68.34 ± 6.44	< 0.0001
PIR	2.71 ± 1.53	2.08 ± 1.16	3.21 ± 1.51	< 0.0001
Sedentary time (min)	435.30 ± 192.74	347.03 ± 127.39	338.06 ± 162.28	< 0.0001
CGS (kg)	48.41 ± 11.12	48.13 ± 10.41	50.63 ± 9.01	0.0001
BMI (kg/m^2^)	30.25 ± 6.97	31.32 ± 6.43	27.76 ± 6.07	< 0.0001
Waist circumference (cm)	101.67 ± 13.39	105.36 ± 15.51	96.08 ± 13.66	< 0.0001
Alcohol frequency (times/years)	2.70 ± 3.02	3.05 ± 2.13	3.17 ± 3.29	0.0168
Sleep duration (hours)	7.23 ± 1.43	6.71 ± 1.04	7.07 ± 1.23	0.0130
Cognitive function (score)	0.14 ± 0.83	0.54 ± 0.80	0.50 ± 0.75	< 0.0001
IR (score)	0.18 ± 1.04	0.48 ± 0.80	0.43 ± 0.92	< 0.0001
DR (score)	0.12 ± 1.06	0.63 ± 0.95	0.40 ± 0.89	< 0.0001
AF (score)	0.03 ± 0.98	0.56 ± 1.52	0.45 ± 0.99	< 0.0001
DSST (score)	0.24 ± 1.03	0.50 ± 0.74	0.71 ± 0.96	< 0.0001
**Categorical scalar (%)**				
**Race**				0.2879
Mexican American	3.54	4.53	2.88	
Other Hispanic	3.87	75.53	3.84	
Non-Hispanic White	77.14	7.43	81.07	
Non-Hispanic Black	10.62	12.51	7.53	
Other	4.84	0	4.68	
**Education level (years)**				0.0001
< 9	19.94	5.77	12.23	
≥9	80.06	94.23	87.77	
**Smoking status**				0.3535
Yes	41.96	53.17	40.35	
No	58.04	46.83	59.65	
**Sleep disorders**				0.8011
Yes	37.75	32.39	36.80	
No	62.25	67.61	63.20	
**Depressive symptoms**				0.0001
Yes	13.02	15.40	6.41	
No	86.98	84.60	93.59	
**Diabetes**				0.0001
Yes	21.11	26.97	12.71	
No	75.87	72.26	82.23	
Boundary	3.02	0.77	5.06	

M ± SD, mean ± standard deviation; PIR, Ratio of family income to poverty; CGS, Combined grip strength; IR, immediate recall; AF, Animal fluency; DR, delayed recall; DSST, Digit symbol substitution test.

### 3.2 Positive relationship between physical activity and cognitive function

The regression analysis outcomes, as delineated in Table 2, explore the association between PA, treated as both a continuous variable and segmented into categories (inactive, WW, RE), and CF among elderly female participants. The findings reveal that with every additional hour of PA, there is a significant increment of 0.003 in the overall CF scores (β-PA: 0.003, 95% CI: 0.000–0.006, *P* = 0.03143). Our results indicate that participants categorized within the WW and RE exhibit markedly better cognitive performance compared to those in the inactive group (β-WW: 0.451, 95% CI: 0.216–0.685, *P* = 0.00017; β-RE: 0.153, 95% CI: 0.085–0.221, *P* = 0.00001). These results underscore the positive correlation between increased PA levels and enhanced CF in this demographic.

**Table 2:** Association between physical activity and cognitive function.

	**Model 1 β (95%CI)**	**Model 2 β (95%CI)**	**Model 3 β (95%CI)**
PA (hours)	0.009 (0.006, 0.013) < 0.00001	0.004 (0.001, 0.007) 0.00992	0.003 (0.000, 0.006) 0.03143
Inactive	0 (reference)	0 (reference)	0 (reference)
WW	0.401 (0.108, 0.694) 0.00731	0.447 (0.210, 0.684) 0.00023	0.451 (0.216, 0.685) 0.00017
RE	0.359 (0.278, 0.441) < 0.00001	0.169 (0.101, 0.236) < 0.00001	0.153 (0.085, 0.221) 0.00001
*P* for trend	< 0.001	< 0.001	< 0.001
*R^2^*	0.047	0.354	0.382

WW, Weekend Warrior; RE, Regular exercise; PA, Physical activity; CF, Cognitive function; Model 1, Unadjusted Variables. Model 2, Race, age, education level, ratio of family income to poverty were adjusted. Model 3, Race, age, education level, ratio of family income to poverty, alcohol frequency, waist circumference, BMI, smoking status, diabetes, depressive symptom, sleep disorders were adjusted.

### 3.3 Association between PA intensity and CF

Table 3 presents the regression analysis between the intensity of PA and CF. The results indicate a progressive increase in CF scores with escalating PA intensity compared to participants who exclusively engaged in MPA. Specifically, the trend analysis reveals a significant positive correlation (*P* for trend < 0.001), indicating that as PA intensity increases, so does the cognitive performance. In detail, participants with MPA-dominated activities show an increase in cognitive scores (β-MPA-dominated: 0.16, 95% CI: 0.02–0.09, *P* = 0.0208), and this enhancement is more pronounced in individuals with VPA-dominated activities (β-VPA-dominated: 0.21, 95% CI: 0.09–0.34, *P* = 0.0011). Participants engaging exclusively in Total VPA exhibit the highest increment in CF scores, although this result approaches the threshold of statistical significance (β-Total VPA: 0.31, 95% CI: −0.01–0.63, *P* = 0.0566).

**Table 3:** Association between physical activity intensity and cognitive function.

	**Model 1 β (95%CI)**	**Model 2 β (95%CI)**	**Model 3 β (95%CI)**
Total MPA	0 (reference)	0 (reference)	0 (reference)
MPA-dominated	0.51 (0.35, 0.66) < 0.0001	0.12 (−0.01, 0.25) 0.0709	0.16 (0.02, 0.29) 0.0208
VPA-dominated	0.54 (0.39, 0.69) < 0.0001	0.24 (0.11, 0.37) 0.0002	0.21 (0.09, 0.34) 0.0011
Total VPA	0.50 (0.10, 0.89) 0.0141	0.35 (0.03, 0.68) 0.0336	0.31 (−0.01, 0.63) 0.0566
*P* for trend	< 0.001	< 0.001	< 0.001

PA, Physical activity; CF, Cognitive function; Total MPA, All physical activities are of moderate intensity; MPA-dominated, Physical activity is predominantly moderate-intensity physical activity; VPA-dominated, Physical activity is predominantly vigorous-intensity physical activity; Total VPA, All physical activities are of vigorous-intensity; Model 1, Unadjusted Variables. Model 2, Race, age, education level, ratio of family income to poverty were adjusted. Model 3, Race, age, education level, ratio of family income to poverty, alcohol frequency, waist circumference, BMI, smoking status, diabetes, depressive symptom, sleep disorders were adjusted.

### 3.4 Smoothed curves and threshold effect analysis

**Figure 2:**
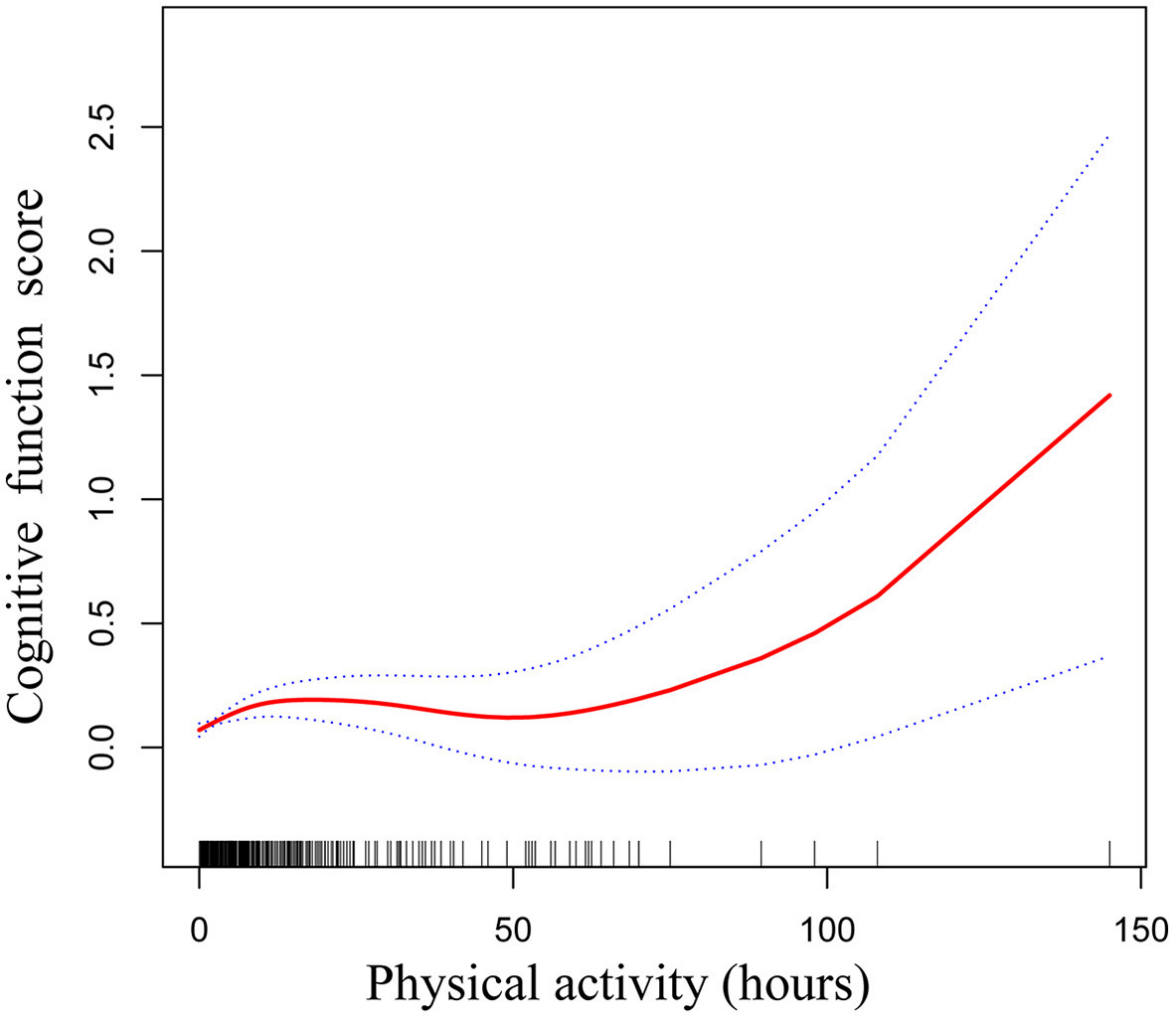
Smoothed curve of physical activity and cognitive function.

Tables 4, 5 elucidate the threshold effect analysis concerning the relationship between PA levels and intensity with CF. The analysis identifies a pivotal threshold for PA at 2.5 h per week. Below this inflection point, every additional hour of PA per week is associated with a significant increase in overall cognitive scores by 0.08. However, beyond 2.5 h per week, the relationship between PA and CF does not exhibit statistical significance. The smoothing curve between PA and CF is shown in Figure 2.

**Table 4:** Threshold effect analysis of physical activity on cognitive function.

	**β (95%CI)**
One-line linear regression model	0.00 (0.00, 0.01) 0.0314
**Two-piecewise linear regression model**	
Inflection point (K)	2.5
PA < K (hours/week)	0.08 (0.04, 0.11) < 0.0001
PA ≥ K (hours/week)	−0.00 (−0.00, 0.00) 0.8153
Log-likelihood ratio	< 0.001

**Table 5:** Threshold effect analysis of physical activity intensity on cognitive function.

	**β (95%CI)**
One-line linear regression model	0.30 (0.15, 0.46) < 0.0001
**Two-piecewise linear regression model**	
Inflection point (K)	63%
PA intensity < K	0.26 (0.04, 0.48) 0.0223
PA intensity ≥ K	0.55 (−0.29, 1.39) 0.2015
Log-likelihood ratio	0.560

Regarding the intensity of PA, the relationship with CF is more accurately depicted by a one-line linear regression model (see Table 5). The result demonstrates that with each one percent increase in PA intensity, there is a corresponding enhancement in the overall CF scores of participants by 0.30 (β = 0.30, 95% CI: 0.15–0.46, *P* < 0.0001), suggesting a linear and positive association between the intensity of PA and cognitive health outcomes.

### 3.5 Subgroup analysis

Upon conducting interaction analyses stratified by depressive symptoms, level of education, BMI, and combined grip strength, no significant interaction effects were observed across the subgroups (*P* > 0.05, Table 6). This lack of significant interactions reinforces the robustness of the results obtained in our research.

**Table 6:** Subgroup analysis of the relationship between physical activity and cognitive function.

	**Model 1 β (95%CI)**	**Model 2 β (95%CI)**	**Model 3 β (95%CI)**
PA (hours)	0.009 (0.006, 0.013) < 0.00001	0.004 (0.001, 0.007) 0.00992	0.003 (0.000, 0.006) 0.03143
**Stratified by depressive symptom**			
Yes	0.01 (−0.00, 0.02) 0.1052	0.01 (−0.00, 0.02) 0.2597	0.01 (−0.00, 0.02) 0.1776
No	0.01 (0.01, 0.01) < 0.0001	0.00 (−0.00, 0.01) 0.0525	0.00 (−0.00, 0.01) 0.0732
*p* for interaction	0.7592	0.5700	0.3835
**Stratified by education level**			
< 9 years	0.00 (−0.00, 0.01) 0.3417	0.00 (−0.01, 0.01) 0.9081	0.00 (−0.01, 0.01) 0.7033
>9 years	0.01 (0.00, 0.01) < 0.0001	0.00 (−0.00, 0.01) 0.0559	0.00 (−0.00, 0.01) 0.0539
*p* for interaction	0.4646	0.5612	0.7468
**Stratified by combined grip strength**			
High level	0.01 (0.00, 0.01) 0.0012	0.00 (−0.00, 0.01) 0.1326	0.00 (−0.00, 0.01) 0.1862
Low level	0.01 (0.00, 0.01) 0.0051	0.00 (−0.00, 0.01) 0.1178	0.00 (−0.00, 0.01) 0.1334
*p* for interaction	0.7020	0.6835	0.6446
**Stratified by BMI**			
Normal	0.01 (0.01, 0.02) 0.0001	0.00 (−0.00, 0.01) 0.0949	0.00 (−0.00, 0.01) 0.2917
Overweight	0.01 (0.00, 0.01) 0.0057	0.00 (−0.00, 0.01) 0.4678	0.00 (−0.00, 0.01) 0.6476
Obesity	0.01 (0.00, 0.02) 0.0058	0.00 (−0.00, 0.01) 0.0987	0.00 (−0.00, 0.01) 0.0740
*p* for interaction	0.5346	0.6601	0.6011

PA, Physical activity; CF, Cognitive function; BMI, Body mass index. Model 1, Unadjusted Variables. Model 2, Race, age, education level, ratio of family income to poverty were adjusted. Model 3, Race, age, education level, ratio of family income to poverty, alcohol frequency, waist circumference, BMI, smoking status, diabetes, depressive symptom, sleep disorders were adjusted.

## 4 Discussion

Through our cross-sectional analysis of NHANES 2011–2014 data, we confirmed a positive association between PA and CF among elderly women. Notably, even participants identified as WW exhibited a correlation with enhanced CF in this cohort. Additionally, our findings underscore the particular relevance of intensity of PA as a significant determinant of superior cognitive performance in elderly women. This study underscores the importance of PA, irrespective of its frequency, in maintaining and potentially improving cognitive health in the aging female population.

Our findings are consistent with existing literature that underscores the beneficial effects of PA on CF in the elderly (Gu et al., 2020; López-Bueno et al., 2023; Feter et al., 2024), reinforcing the notion that an active lifestyle contributes positively to cognitive health. However, our study extends previous research by delineating specific patterns of PA, particularly highlighting the WW model, which has not been extensively explored in relation to cognitive outcomes in older women. Contrary to some studies that suggest only regular, consistent exercise yields cognitive benefits (López-Bueno et al., 2023; Makino et al., 2024), our results indicate that engaging in PA concentrated in fewer days (WW pattern) is also associated with improved CF. This distinction offers a new perspective on the flexibility of exercise scheduling and its potential cognitive benefits. Additionally, while previous research has predominantly focused on the volume of PA (Piercy et al., 2018; Abdullahi et al., 2024), our study uniquely emphasizes the significance of activity intensity, suggesting that MVPA has a more pronounced association with cognitive health compared to lower intensity activities. This nuance adds to the growing body of evidence advocating for the inclusion of more vigorous activities in exercise recommendations for the elderly.

The observation that elderly women categorized as WW exhibit higher cognitive levels compared to their inactive counterparts, and comparably favorable outcomes to those engaging in RE, invites exploration into the underlying mechanisms. One potential explanation could involve the intensity and consolidation of PA into fewer, longer sessions, which might induce more pronounced acute physiological responses known to benefit brain function, such as increased cerebral blood flow (Augusto-Oliveira et al., 2023), enhanced neurogenesis (Ryan and Kelly, 2016; Ryan and Nolan, 2016), and improved depressive symptom (Liang et al., 2023; Wang et al., 2024) and sleep quality (Druzian et al., 2024). Additionally, the WW may align better with the lifestyles of older adults, offering a more achievable and sustainable approach to PA, which in turn could lead to more consistent long-term engagement (Dos Santos et al., 2022; O'Donovan et al., 2024). This pattern of activity might also mitigate the effects of sedentary behavior accumulated over the week (Khurshid et al., 2023), countering cognitive decline more effectively than previously assumed (Öztürk et al., 2023; Raichlen et al., 2023). Furthermore, the psychological benefits of fulfilling exercise recommendations within a shorter time frame could contribute to reduced stress and improved mental health (Agudelo et al., 2014; Gujral et al., 2024), thereby indirectly benefiting CF. These hypotheses align with the concept that not only the quantity but also the quality and timing of exercise can play critical roles in influencing cognitive health.

On the other hand, our findings clearly demonstrate that MVPA confers greater cognitive benefits in elderly women than lower intensity physical activities. This enhancement in cognitive function can be attributed to several interrelated physiological and neurobiological mechanisms. MVPA significantly increases cerebral blood flow, essential for delivering nutrients and oxygen to the brain and facilitating the clearance of neurotoxic waste products, potentially preventing neurodegenerative diseases such as Alzheimer's (Nguyen et al., 2023; Raji et al., 2024). Additionally, MVPA stimulates the release of neurotrophic factors like brain-derived neurotrophic factor (BDNF), which supports neuron survival and growth, and enhances synaptic plasticity, crucial for learning and memory (Mata et al., 2010; Sanders et al., 2019). Furthermore, the metabolic improvements associated with MVPA, including better glucose regulation and lipid profiles, are vital for cognitive health (Valentine et al., 2007). Psychological benefits, such as the alleviation of depression and anxiety symptoms through the mood-enhancing effects of physical activity, also contribute to its cognitive advantages (Kreppke et al., 2024; Li et al., 2024). Intriguingly, the WW group, which engages in infrequent but highly intense exercise sessions, may experience an additional cognitive boost. This could be due to the heightened acute physiological responses elicited by the concentrated bouts of intense physical exertion, enhancing cerebral perfusion and neuroprotective responses more robustly than more frequent, less intense sessions (Khurshid et al., 2023). These findings underscore the importance of considering not only the frequency but also the intensity of exercise in formulating physical activity recommendations to maximize cognitive health benefits and tailor public health strategies to the unique needs and capabilities of the elderly.

Our study has several strengths, including the utilization of a large, nationally representative sample from NHANES 2011–2014, which enhances the generalizability of our findings. Additionally, the application of rigorous statistical analyses, including regression models and threshold effect analysis, allows for a nuanced understanding of the relationship between PA patterns and CF in elderly women. Furthermore, the categorization of PA into distinct patterns, such as WW and RE, alongside the consideration of activity intensity, provides a detailed examination that surpasses many previous studies in this field. However, our research is not without limitations. The cross-sectional design restricts our ability to infer causal relationships between PA and CF; longitudinal studies are necessary to establish causality. Additionally, the reliance on self-reported PA data may lead to reporting biases and inaccuracies. While the GPAQ is a validated instrument, objective measures such as accelerometry could provide a more accurate assessment of PA levels. Although the complex multistage sampling and weighting procedures used in NHANES enhance the representativeness of the sample, the small sample size of the WW group may limit the generalizability of our results to this specific pattern of activity. Additionally, while physical activity is a crucial predictor of cognitive function, it is only one of many factors that contribute to cognitive health. Lastly, our study focused exclusively on elderly women, which, while addressing an important demographic, limits the applicability of our findings to other groups. Future research should explore similar associations in diverse populations and through longitudinal designs to address these limitations.

## 5 Conclusion

Our study has affirmed a positive association between PA and CF in elderly women, particularly with MVPA. Interestingly, our findings also reveal that elderly women engaged in WW and RE regimens exhibit better CF compared to those who are inactive. These insights highlight the critical role that even intermittent, intensive PA play in bolstering cognitive health in older women.

## Data availability statement

The original contributions presented in the study are included in the article/supplementary material, further inquiries can be directed to the corresponding author.

## Ethics statement

Ethical review and approval was not required for the current study in accordance with the local legislation and institutional requirements. Written informed consent for participation was not required for this study in accordance with the national legislation and the institutional requirements.

## Author contributions

JW: Conceptualization, Data curation, Writing – original draft, Writing – review & editing. PQ: Data curation, Investigation, Methodology, Visualization, Writing – original draft. MLiu: Investigation, Visualization, Writing – original draft. WY: Data curation, Investigation, Visualization, Writing – original draft. MLi: Data curation, Visualization, Writing – original draft. YL: Supervision, Writing – review & editing.
